# Acute Low Force Electrically Induced Exercise Modulates Post Prandial Glycemic Markers in People with Spinal Cord Injury

**DOI:** 10.3390/jfmk7040089

**Published:** 2022-10-17

**Authors:** Michael A. Petrie, Amy L. Kimball, Richard K. Shields

**Affiliations:** Department of Physical Therapy and Rehabilitation Science, Carver College of Medicine, The University of Iowa, Iowa City, IA 52242, USA

**Keywords:** hyperinsulinemia, glucose tolerance, neuromuscular electrical stimulation, paralysis

## Abstract

Regular exercise involves daily muscle contractions helping metabolize up to 70% of daily ingested glucose. Skeletal muscle increases glucose uptake through two distinct pathways: insulin signaling pathway and muscle contraction mediated AMPK pathway. People with paralysis are unable to contract their muscles which atrophy, transform into insulin resistant glycolytic muscle, and develop osteoporosis. Our goal is to determine if low force electrically induced exercise (LFE) will modulate the post prandial insulin and glucose response in people with and without spinal cord injury (SCI). 18 people with SCI and 23 without SCI (Non-SCI) participated in an assessment of metabolic biomarkers during passive sitting (CTL) and a bout of LFE delivered to the quadriceps/hamstring muscle groups after a glucose challenge. Baseline fasting insulin (*p* = 0.003) and lactate (*p* = 0.033) levels were higher in people with SCI, but glucose levels (*p* = 0.888) were similar compared to the non-SCI population. After 1-h of muscle contractions using LFE, heart rate increased (*p* < 0.001), capillary glucose decreased (*p* = 0.004), insulin decreased (*p* < 0.001), and lactate increased (*p* = 0.001) in the SCI population. These findings support that LFE attenuates certain metabolic blood biomarkers during a glucose challenge and may offer a lifestyle strategy to regulate metabolic responses after eating among people with SCI.

## 1. Introduction

Regular muscle contractions are associated with greater metabolic control [1,2]. Daily physical activity recommendations are well established for people with intact central nervous systems [3]. However, people with spinal cord injury (SCI), who are paralyzed, not only lose extensive volitional movement, but also lose over 50% of their skeletal muscle mass from atrophy [4,5,6]. We now understand that skeletal muscle is a major endocrine organ that can metabolize up to 70% of glucose after a meal [7]. While people with spinal cord injury often have control of their upper extremities, the upper extremities alone are incapable of effectively impacting post prandial metabolic biomarkers [8]. Recent work supports the need for alternative “lifestyle” [6] strategies that engage large muscle groups to impact acute metabolic regulation [8]. Our goal is to understand if electrically induced exercise of paralyzed muscle will acutely reduce carbohydrate induced insulin spikes among people with chronic SCI.

**Figure 1:**
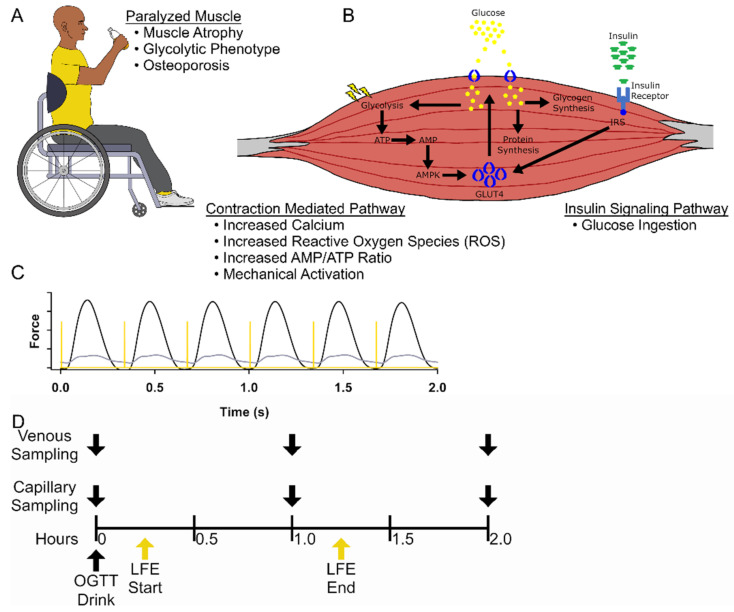
Schematic of experimental design. (**A**) People with a spinal cord injury (SCI) use wheelchairs to enable locomotion and increased independence; however develop osteoporosis, muscle atrophy, highly glycolytic muscle fibers that become less responsive to insulin-dependent glucose uptake, leading to a hyperinsulinemia. (**B**) Representative schematic of the mechanisms utilized by skeletal muscle to control blood glucose concentration. Our LFE bypasses the insulin-dependent mechanism to promote the translocation of GLUT4 to the sarcolemma through the activation of AMPK. (**C**) A representative example from a force tracing of quadriceps muscles at the start (black) and end (gray) of a bout of LFE with the associated stimulus pulse (yellow). (**D**) Schematic of study timeline of venous blood sampling and capillary blood sampling during the control session and the LFE session that was started 15-min after consuming a 75 g glucose beverage.

Skeletal muscle contractions increase the rate of glucose uptake, independent of insulin, through the AMP-activated protein kinase (AMPK) GLUT-4 pathway [1,9,10] and forms the basis for the lifestyle recommendation that we should “be active after a meal” rather than sedentary [11,12]. People with SCI, who have atrophied skeletal muscle, develop significant hyperinsulinemia [13], insulin resistance [14], and eventually diabetes [15,16]. People with hyperinsulinemia in response to a glycemic load are categorized as “hyper-secretors”, a trait that is now recognized as a precursor to the development of type II diabetes [17]. Inactivity from paralysis after SCI also leads to tissue adaptations that increase visceral adiposity [18,19], decrease bone density [20,21,22], reduce muscle mass [4,23], decrease overall systemic metabolic flexibility [13,24,25], and transitions paralyzed skeletal muscle from an “insulin sensitive” oxidative organ to an atrophied “insulin insensitive” glycolytic organ [23,26] (Figure 1A,B). Together, these adaptations foster an unstable systemic metabolism supported by elevated biomarkers for inflammation (C-reactive protein), abnormal lipid profiles, and abnormal glycemic control (glucose, insulin, lactate) in people with paralysis [13,14,27].

Neuromuscular electrical stimulation, also known as “functional electrical stimulation”, has been used to “turn on” paralyzed muscle for many years [28,29]. Neuromuscular electrical stimulation provides short bursts of electrical current to peripheral nerves to trigger an action potential over the muscle sarcolemma, into the t-tubule to trigger calcium release from the sarcoplasmic reticulum and induce excitation-contraction coupling and cross-bridge cycling [30]. The bioenergetics of excitation-contraction coupling, and skeletal muscle cross-bridge cycling involves the conversion of adenosine triphosphate (ATP) to adenosine diphosphate (ADP) and adenosine monophosphate (AMP) [30]. The AMP/ATP ratio signals the AMPK to GLUT4 pathway to increase glucose movement into the skeletal muscle without relying on insulin [31]. Other stimuli of the AMPK to GLUT4 pathway are calcium release [32], accumulation of reactive oxygen species (ROS) [33,34], and muscle mechanical stress [35] (Figure 1B). Because the bioenergetics of electrically induced muscle activity are similar to volitionally induced muscle contraction [36], a timely dose of electrically induced exercise, after a glucose challenge, may offer a strategy to blunt the insulin spikes that are customary among people with SCI. Previous studies in people with type II diabetes support that electrically induced muscle exercise reduces blood glucose acutely [37,38] and chronically [38], supporting the need for this study. In addition, we focus on several biomarkers, including insulin, to better understand the response that electrically induced exercise has on people with SCI.

While it appears logical that postprandial electrically induced muscle contractions may minimize insulin peaks among people with paralysis, there are several reasons why this needs to be scientifically explored. First, the paralyzed skeletal muscle “organ” is nearly 50% atrophied so there is an overall reduction in overall muscle mass [4]. Second, the paralyzed organ transforms to fast glycolytic muscle [23,39], so it fatigues quickly, builds up lactate, and cannot sustain long duration activity due to decreased oxidative phosphorylation from the loss of mitochondria [23,40,41]. Lastly, paralyzed muscle tendons insert into a skeletal system that loses nearly 50% of its bone mineral density [21,26,42,43,44] (osteoporosis) putting the bone at fracture risk when electrically generating high tetanic muscle forces [45]. Because of these constraints, electrically induced exercise must be delivered at a low force. To be effective, the low force will need to impact signaling pathways that involve AMP/ATP, ROS, calcium, and lactate (Figure 1A,B), all pathways that trigger GLUT4 transport of glucose into muscle.

Recently, we discovered that by using a low frequency (3 Hz) and a high intensity stimulation, we could recruit most of the paralyzed muscle [41], but at a very low force level (<10% of tetanic max). This protocol minimized the mechanical load to the underlying osteoporotic bone [41], but also turned on major genomic and epigenomic oxidative signaling pathways [6,41,46], and, with training begins to convert the organ from a glycolytic muscle to a more endurant muscle [6,47]. Further, we have shown that bilateral electrically induced exercise using low frequencies increases oxygen consumption and energy expenditure for people with and without SCI [18]. In this study, we propose to examine if this same LFE protocol will regulate metabolic biomarkers, like insulin, after a glucose challenge.

The purpose of this study is to assess if a novel low force electrically induced exercise (LFE) modulates the glucose, insulin, lactate, and heart rate (HR) responses following an oral glucose challenge among people with and without SCI. We expect that the LFE protocol will attenuate the insulin response with a secondary influence on glucose after the glucose challenge in people with and without SCI. In the end, these findings will inform us as to whether low force electrically induced exercise offers a plausible lifestyle strategy to blunt the hypersecretion of insulin following a glucose challenge among people with SCI.

## 2. Materials and Methods

### 2.1. Glycemic, Inflammatory, and Lipid Profile

Forty-one individuals (18 with and 23 without SCI) were enrolled in this study (Table 1). All participants with SCI (7 people with paraplegia and 11 people with tetraplegia) were classified as chronic with average time since injury of 10.7 ± 8.6 years. Additionally, all people with SCI had an upper motor neuron lesion resulting in a complete loss of movement and sensation below the lesion level, but intact peripheral nerves to the paralyzed lower extremity muscles (American Spinal Cord Injury Association Impairment Score A). People with SCI were excluded if they had recently trained with electrical stimulation, were intolerant to electrical stimulation due to autonomic dysreflexia, had a recent history of lower limb fractures, peripheral nerve injuries to the lower extremity, pressure ulcers, muscle injury, or had systemic illness. People without SCI were recruited from a sample of convenience and were excluded if they recently trained volitionally or had any systemic illness or chronic disease. Additionally, people that have previously been diagnosed with type 2 diabetes, or actively taking a pharmaceutical to address elevated blood glucose, were excluded from participation. All participants were asked to not perform any moderate to vigorous physical activity 48 h prior to participation. All participants provided written informed consent approved by the institutional review board at the University of Iowa in accordance with the Declaration of Helsinki.

### 2.2. Experimental Design

Each participant had a control (CTL) session and a low force exercise (LFE) session using electrical muscle stimulation for 1-h during a 2-h oral glucose tolerance test (OGTT). Prior to the start of each session, participants were required to fast for at least 4-h. Each session consisted of a fasting baseline (00HR) venous blood draw, finger prick for capillary blood, and radial pulse. Participants were also instrumented with a heart rate activity monitor (FirstBeat Technologies Ltd., Jyväskylä, Finland) for the duration of the session. Participants consumed a 10-ounce 75 g dextrose drink (Trutol, Thermo Scientific Inc., Waltham, MA, USA) to begin the 2-h OGTT. Capillary blood, venous blood, and a radial pulse were obtained at 1-h (01HR; 9 with SCI; 17 without SCI) and 2-h (02HR; 18 with SCI; 23 without SCI) after the dextrose ingestion. Because the first 9 participants with SCI showed a robust effect size at the 01HR time, we were able to reduce the risk of venipuncture from three to two (00HR and 02HR) for the remaining 9 participants with SCI. Control and LFE sessions occurred on non-consecutive days with at least 5 days between sessions.

During control and LFE sessions, participants with SCI remained seated in their personal wheelchairs, and non-SCI participants sat in a padded manual wheelchair. To minimize energy expenditure, participants were instructed to limit movement during data collection. The LFE protocol session consisted of a 1-h bout of 3Hz electrical stimulation delivered bilaterally to the hamstrings and quadriceps, which started 15 min after the start of the OGTT (Figure 1).

### 2.3. Electrical Stimulation

For the LFE session, 4 pairs of self-adhesive 7 × 13 cm rectangular carbon electrodes (Dura-Stick Plus, DJO, LLC, Vista, CA, USA) were adhered to the skin over the quadriceps and hamstring muscles motor points on each leg. Muscle selection was based on ease of access, size, clinical importance, and likelihood to alter skeletal muscle metabolism. Anteriorly, the proximal quadriceps electrodes were placed close to the inguinal crease and over the most palpable border of the vastus lateralis. Care was taken to keep the medial border of the proximal electrode lateral to the adductor muscle group. The distal quadriceps electrodes were placed close the knee with the distal border of the distal border of the electrode proximal to the musculotendinous junction quadriceps and the knee. Posteriorly, the proximal hamstring electrodes were placed near the gluteal fold with the medial electrode border lateral to the adductor muscle group. The distal hamstring electrodes were placed over the semimembranosus and biceps femoris muscles proximal to the popliteal fossa. Legs were secured below the knee to minimize knee extension or flexion movement.

Stimulus pulses (3 Hz) were delivered using a constant current muscle stimulator (DS7A, Digitimer, Welwyn Garden City, Hertfordshire, UK) controlled by a computer. Stimulus pulses had a 200 μs stimulus pulse width with an intensity range of 100–200 mA to the quadriceps and 50–100 mA to the hamstrings. Stimulating with a frequency of 3 Hz prevents skeletal muscle summation but still challenges the calcium release and contractile filaments by inducing significant fatigue [41] (Figure 1C). Stimulation intensity was selected for near maximal recruitment of the quadriceps and hamstrings as previously established for people with SCI [41]. We used the maximal intensity tolerated for people without SCI. To achieve the near maximal muscle recruitment, the stimulation current was systematically increased until there was no perceived increase in force development with an increase in stimulation current for people with SCI. For the non-SCI participants, the stimulation intensity was systematically increased to tolerance. Overall, clear strong contractions were induced in both populations although the Non-SCI participants tolerated lower current (~70 mA) than those with SCI (>100 mA) Higher current is necessary for people with SCI because of the thickness of the adipose layer and edema from inactivity.

### 2.4. Blood Collection and Analysis

Venous blood was collected via an antecubital, forearm, or dorsal metacarpal venipuncture with a 23G or 21G butterfly needle. A separate venipuncture occurred for each blood collection. Blood was collected in 5 mL serum vacutainer tubes (Becton, Dickinson and Company, Franklin Lakes, NJ, USA). Blood samples were immediately centrifuged to separate hematocrit from serum for immediate analysis on an automated blood chemistry analyzer (BS200, Shenzhen Mindray Bio-Medical Electronics Co., Ltd., Shenzhen, China) for all biomarkers. Glucose (GLU) and lactate (LAC) concentrations were quantified from venous serum using reagents from MedTest Dx and Pointe Scientific (MedTest Holdings, Canton, MI, USA) according to manufacturer guidelines. Insulin (INS) concentrations were quantified using reagents from Kamiya Biomedical Company (Kamiya Biomedical Company, Seattle, WA, USA) according to manufacturer guideline. A venous glucose to insulin ratio (G:I Ratio) was calculated as an estimate of insulin sensitivity by dividing the venous glucose concentration by the venous insulin concentration. Capillary blood was obtained using the finger prick method to quantify capillary glucose (cGLU) concentration using the Contour^®^ USB blood glucose monitoring system (Bayer HealthCare, Tarrytown, NY, USA). The capillary glucose area under the curve (cAUC) during the OGTT was calculated.

### 2.5. Statistical Analysis

Statistical analysis was conducted using SigmaPlot 11.0 (San Jose, CA, USA). Descriptive statistics were reported as means (±SD). Independent *t*-tests were performed to determine baseline differences between SCI and non-SCI participants for age, height, weight, body mass index (BMI), resting heart rate (HR), and fasting blood biomarkers (GLU, INS, G:I Ratio, and LAC). A mixed model analysis of variance was used to test for differences in heart rate and blood biomarkers (GLU, INS, G:I Ratio, LAC, cAUC, HR) between populations (SCI versus non-SCI) and sessions (CTL versus LFE) at the start (00HR), after stimulation (01HR), and at the end (02HR) of the OGTT. The assumption of normality was tested using the Shapiro–Wilk procedure and the assumption of equal variance was tested to ensure parametric statistical procedures were appropriate for all comparisons. All post hoc testing was performed using the Holm-Sidek procedure. All tests were performed using a significance level of *p* < 0.05. Adjustments for multiple comparisons were included in our analytical model.

We required a sample size of 12, 12, 6, 24, and 6 with a power of 0.80 to detect change for venous insulin, G:I ratio, lactate, capillary glucose, and heart rate, respectively, between LFE and CTL at 1 h after glucose ingestion. We required a sample size of 44, 8, 14, 4, and 30 with a power of 0.80 to detect an interaction between population (SCI, nonSCI) and session (CTL, LFE) for insulin, G:I ratio, lactate, and heart rate, respectively. Accordingly, given the variance of the dependent measures, we were slightly underpowered to detect change for glucose after 1 h.

## 3. Results

### 3.1. Glycemic, Inflammatory, and Lipid Profile

Several baseline glycemic, inflammatory, and lipid biomarkers were different between people with and without SCI, except for fasting glucose, as summarized in Table 1. People with SCI had higher fasting insulin (*p* = 0.003), lactate (*p* = 0.033), c-reactive protein (CRP) (*p* < 0.001), uric acid (UA) (*p* = 0.029), alkaline phosphatase (ALP) (*p* < 0.001), and low-density lipoprotein (LDL) (*p* = 0.006) as compared to the non-SCI population. People with SCI also had lower glucose to insulin ratios (*p* = 0.002) and lower high-density lipoprotein (HDL) (*p* < 0.001) compared to non-SCI. Fasting glucose was not different between SCI and non-SCI (*p* = 0.89) supporting that the SCI population was generally healthy and still able to maintain glucose homeostasis, albeit via the elevated insulin levels as compared to people without SCI (Table 1).

### 3.2. Baseline Venous Blood Biomarkers for Control and LFE Sessions

**Figure 2:**
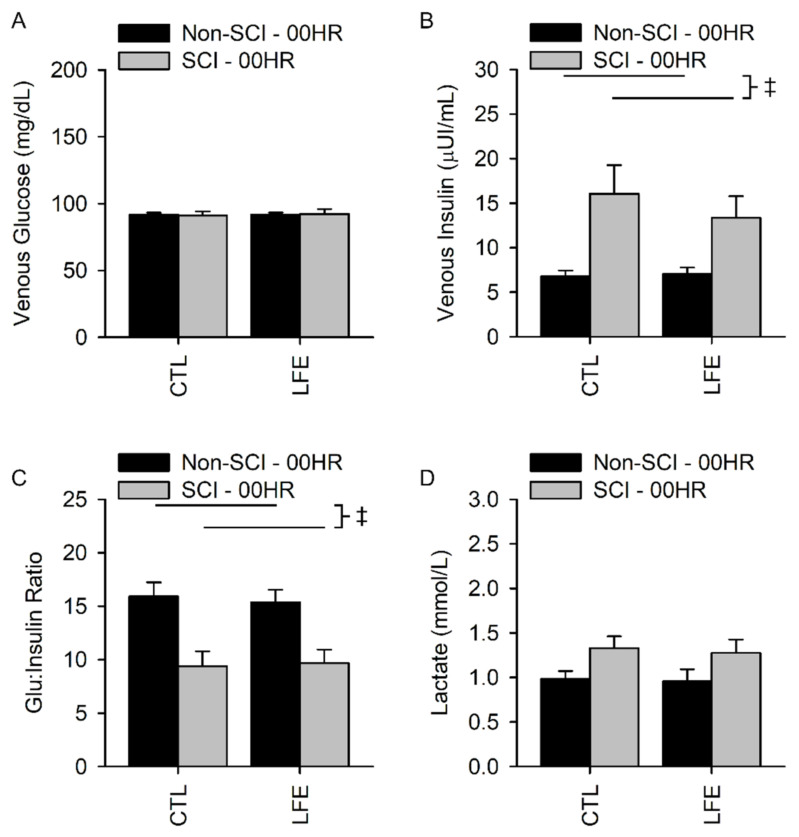
Fasting baseline venous blood biomarkers. There was no difference for fasting serum glucose (**A**) or Lactate (**D**) between people with and without SCI and during the CTL or LFE sessions; but there was a significant difference between insulin (**B**) and the glucose to Insulin ratio (**C**) between SCI and non-SCI. ‡ Indicates a significant difference (*p* ≤ 0.05) of the indicated main effect and no significant interaction between session and population.

Fasting baseline venous glucose levels (GLU) for the control sessions in SCI and non-SCI populations were 91.2 ± 12.3 and 91.6 ± 7. mg/dL, respectively. Fasting baseline venous glucose levels for the LFE sessions in SCI and non-SCI populations were 92.3 ± 14.5 and 91.6 ± 7.9 mg/dL, respectively, showing excellent reproducibility across sessions. There was no interaction between population (SCI versus non-SCI) and session (CTL versus LFE) for fasting baseline venous glucose (*p* = 0.39), nor main effect differences for population (*p* = 0.84) or session (*p* = 0.39) (Figure 2A).

Fasting venous insulin levels for the baseline control sessions in SCI and non-SCI populations were 16.0 ± 13.6 and 6.8 ± 3.1 µUI/mL, respectively. Fasting baseline venous insulin levels for the LFE sessions in SCI and non-SCI populations were 13.4 ± 10.0 and 7.0 ± 3.6 µUI/mL, respectively. There was no interaction between population and session for fasting venous insulin (*p* = 0.74), nor a significant main effect for session (*p* = 0.86). As previously indicated, there was a main effect for population, with people with SCI having elevated fasting insulin as compared to people without SCI (*p* < 0.001) (Figure 2B).

The fasting glucose to insulin ratios (G:I ratio) for the control sessions in SCI and non-SCI populations were 9.4 ± 6.0 and 15.9 ± 6.3, respectively. The fasting G:I ratios for the LFE sessions in SCI and non-SCI populations were 9.7 ± 5.2 and 15.4 ± 5.7, respectively. There was no interaction between population and session for fasting G:I ratio (*p* = 0.73), nor a main effect for session (*p* = 0.60). There was a main effect between populations, with people with SCI having significantly lower fasting G:I ratio compared to people without SCI (*p* = 0.001), consistent with reduced insulin sensitivity (Figure 2C).

Fasting venous lactate levels for the baseline control session in the SCI and non-SCI populations were 1.3 ± 0.6 and 1.0 ± 0.4 mmol/L, respectively. Fasting venous lactate levels for the LFE session in SCI and non-SCI populations were 1.3 ± 0.6 and 1.0 ± 0.7 mmol/L, respectively. There was no interaction between population and session for fasting venous lactate (*p* = 0.84), nor a main effect for session (*p* = 0.56). There was a trend of elevated fasting lactate in people with SCI compared to people without SCI (*p* = 0.06) (Figure 2D).

### 3.3. One Hour Venous Blood Biomarkers for Control and LFE Sessions after Glucose Challenge

**Figure 3:**
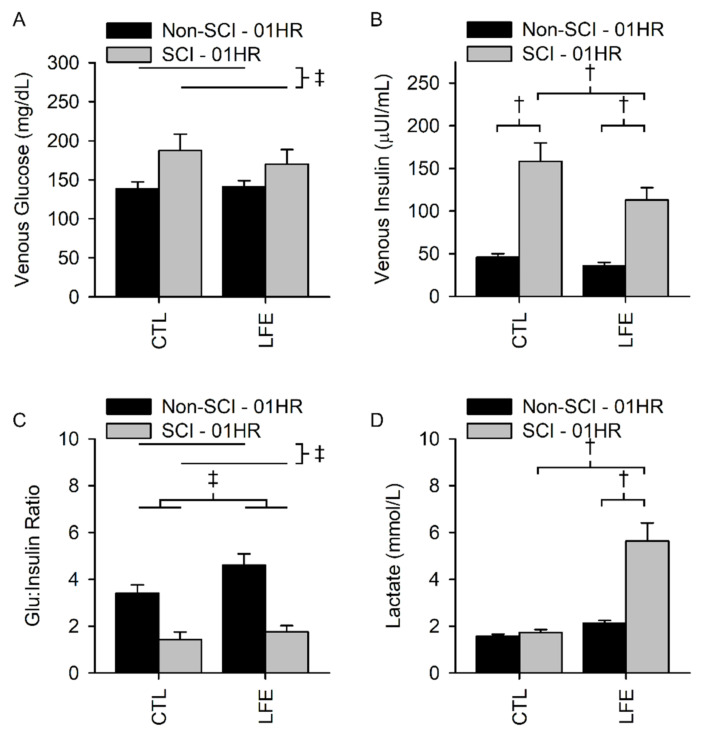
1-h venous blood biomarkers. There was no interaction for glucose (**A**) and the glucose to insulin ratio (**C**). However, people with SCI had a higher glucose response than those without SCI. The glucose to insulin ratio was higher after LFE for both people with and without SCI, though the ratio was lower for people with SCI during both CTL and LFE sessions. There was a significant interaction for insulin (**B**) and lactate (**D**). Insulin was significantly lower after LFE for people with SCI even though their insulin levels were higher than the non-SCI cohort for both the LFE and CTL sessions. Lactate was significantly higher in people with SCI after LFE compared to their control session and compared to the non-SCI cohort after LFE. † Indicates a significant difference (*p* ≤ 0.05) of the indicated simple effect during post hoc testing due to a significant interaction between population and session. ‡ Indicates a significant difference (*p* ≤ 0.05) of the indicated main effect and no significant interaction between session and population.

Venous glucose levels after the glucose challenge for the control sessions in the SCI and non-SCI populations were 187.6 ± 62.6 and 138.5 ± 36.4 mg/dL, respectively. Venous glucose levels for the LFE sessions for SCI and non-SCI populations were 170.2 ± 61.9 and 141.1 ± 32.3 mg/dL, respectively. There was no interaction between population and session (*p* = 0.39) nor a session main effect (*p* = 0.61). There was a population main effect whereby people with SCI had elevated glucose as a result of the challenge as compared to non-SCI (*p* = 0.02) (Figure 3A).

Venous insulin levels after the glucose challenge for the control sessions in the SCI and non-SCI populations were 158.2 ± 64.9 and 45.7 ± 17.6 µUI/mL, respectively. Venous insulin levels for the LFE session for SCI and non-SCI populations were 112.9 ± 48.3 and 36.0 ± 15.9 µUI/mL, respectively, indicating a significant reduction in the amount of insulin required for the glucose challenge. There was an interaction between population and session (*p* = 0.02). Venous insulin levels were lower in the LFE session for people with SCI compared to the control session (*p* < 0.001) but were not different for non-SCI (*p* = 0.34) (Figure 3B).

The G:I ratio for the control sessions for the SCI and non-SCI populations were 1.4 ± 1.0 and 3.4 ± 1.4, respectively. The G:I ratios for the LFE sessions for SCI and non-SCI populations were 1.8 ± 0.9 and 4.6 ± 2.0, respectively. There was no significant interaction between population and session (*p* = 0.11). There was a session main effect whereby the LFE session had a higher G:I ratio compared to the CTL session (*p* = 0.001). There was a population main effect where non-SCI had elevated G:I ratio compared to SCI (*p* < 0.001) (Figure 3C).

Venous lactate levels for the control sessions for SCI and non-SCI populations were 1.7 ± 0.4 and 1.6 ± 0.3 mmol/L, respectively. Venous lactate levels for the LFE sessions for SCI and non-SCI populations were 5.6 ± 2.6 and 2.1 ± 0.5 mmol/L, respectively, indicating that the exercise induced more of the lactate by-product within the SCI population. There was an interaction between population and session (*p* < 0.001). Lactate was elevated during the LFE session compared to the CTL session for people with SCI (*p* < 0.001) but was not different for non-SCI (*p* = 0.12) (Figure 3D).

### 3.4. Two-Hour Venous Blood Biomarkers for Control and LFE Sessions

**Figure 4:**
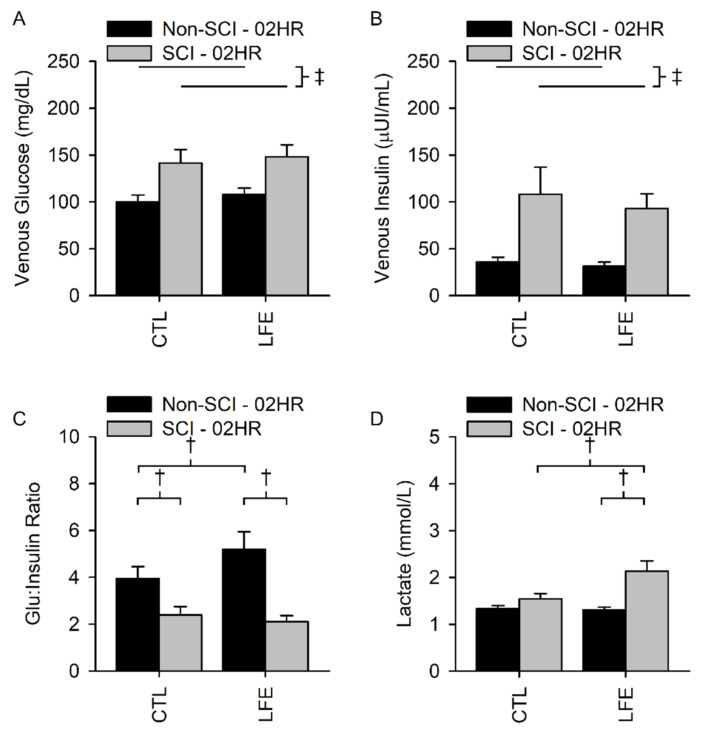
2-h Venous blood biomarkers. There was no interaction for glucose (**A**) and insulin (**B**). However, people with SCI had a higher glucose and insulin response than the non-SCI participants. There was a significant interaction for the glucose to insulin ratio (**C**) and lactate (**D**). People with SCI had a lower glucose to insulin ratio after LFE and during the CTL session compared to the non-SCI participants. Interestingly, there was also an increase in the glucose to insulin ratio for the non-SCI participants after LFE compared to their CTL session. Lactate was significantly higher in people with SCI after LFE compared to their control session and compared to the non-SCI cohort after LFE. † Indicates a significant difference (*p* ≤ 0.05) of the indicated simple effect during post hoc testing due to a significant interaction between population and session. ‡ Indicates a significant difference (*p* ≤ 0.05) of the indicated main effect and no significant interaction between session and population.

Venous glucose (GLU) for the control sessions in SCI and non-SCI populations at the end of the 2-h OGTT were 141.5 ± 58.9 and 100.0 ± 35.0 mg/dL, respectively. Venous glucose for the LFE sessions in SCI and non-SCI populations were 148.1 ± 51.1 and 107.9 ± 32.0 mg/dL, respectively. There was not a significant interaction between population (SCI versus non-SCI) and session (CTL versus LFE) for venous glucose (*p* = 0.65), nor a significant main effect for the session (*p* = 0.062) at the 2-h time point. There was a significant main effect for population whereby people with SCI had a higher glucose compared to those without SCI (*p* = 0.002) (Figure 4A).

Venous insulin for the control sessions in SCI and non-SCI populations at the end of the 2-h OGTT were 107.9 ± 120.3 and 35.8 ± 23.6 µUI/mL, respectively. Venous insulin for the LFE sessions in SCI and non-SCI populations at the end of the 2-h OGTT were 92.8 ± 62.8 and 31.2 ± 21.4 µUI/mL, respectively. There was not a significant interaction between population and session for venous insulin (*p* = 0.78), nor a significant main effect for session (*p* = 0.82). There was a main effect between populations, with people with SCI having significantly elevated insulin compared to people without SCI (*p* < 0.001) (Figure 4B).

The G:I ratio for the control sessions in SCI and non-SCI populations at the end of the 2-h OGTT were 2.4 ± 1.5 and 3.9 ± 2.4, respectively. The G:I ratio for the LFE sessions in SCI and non-SCI populations after the 2-h OGTT were 2.1 ± 1.0 and 5.2 ± 3.5, respectively. There was a significant interaction between population and session for the G:I ratio (*p* = 0.02) at the 2-h time point. The G:I ratio was significantly higher after LFE compared to the CTL session in people without SCI (*p* = 0.005); however, there was no difference in G:I ratio for people with SCI (*p* = 0.42) (Figure 4C).

Venous lactate for the control sessions in the SCI and non-SCI populations at the end of the 2-h OGTT were 1.5 ± 0.5 and 1.3 ± 0.3 mmol/L, respectively. Venous lactate for the LFE sessions in SCI and non-SCI populations at the end of the 2-h OGTT were 2.1 ± 0.9 and 1.3 ± 0.3 mmol/L, respectively. There was a significant interaction between population and session for venous lactate (*p* = 0.001) at the 2-h time point. Venous lactate was elevated only for people with SCI (*p* < 0.001) after LFE compared to the CTL session, while there was no difference in venous lactate for people without SCI (*p* = 0.80) (Figure 4D).

### 3.5. Capillary Glucose (cAUC) and Heart Rate during Control and LFE OGTT

**Figure 5:**
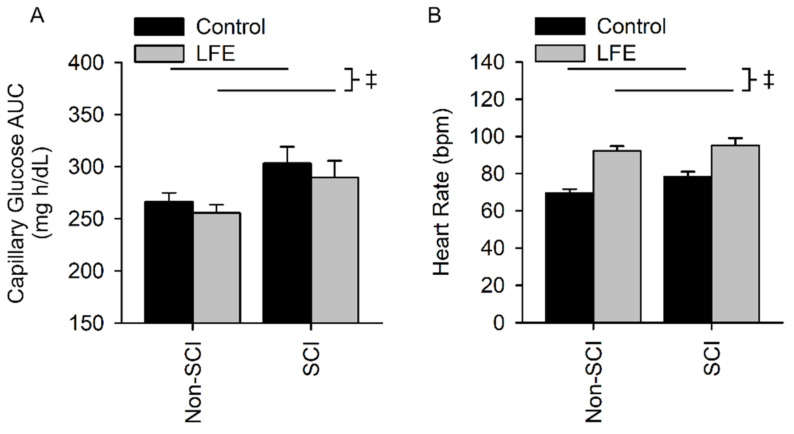
Capillary Glucose Area Under the Curve (cAUC) and Heart Rate. There was no interaction for cAUC (**A**) and maximum heart rate (**B**). However, people with SCI cAUC and maximum heart rate compared to the non-SCI participants. ‡ Indicates a significant difference (*p* ≤ 0.05) of the indicated main effect and no significant interaction between session and population.

Capillary glucose area under the curve(cAUC) for the control sessions in the SCI and non-SCI populations were 303.2 ± 67.3 and 266.4 ± 40.2 mg h/dL, respectively. Fasting capillary glucose levels (cAUC) for the LFE sessions in SCI and non-SCI populations were 289.5 ± 68.0 and 255.6 ± 37.6 mg/dL, respectively. There was no interaction between population and exercise session (*p* = 0.837). There was a difference in cAUC between the control and LFE session but it did not reach a level of significance (*p* = 0.08). However, people with SCI had a significantly higher cAUC compared to people without SCI (*p* = 0.0.027) (Figure 5A).

Heart rate for the control sessions in the SCI and non-SCI populations were 78 ± 11 bpm and 69 ± 11, respectively. Heart rates for the LFE sessions in the SCI and non-SCI populations were 95 ± 16 bpm and 92 ± 12 bpm, respectively. There was no interaction between population and session (*p* = 0.174). There was an exercise session main effect (*p* < 0.001) whereby the heart rate was increased during the LFE session compared to the CTL session (Figure 5B). There was a difference in heart rate between SCI and non-SCI but it did not reach a level of significance (*p* = 0.093).

## 4. Discussion

Hypersecretion of the hormone insulin, either in the fasting or postprandial state, is believed to manifest several years before elevated blood glucose and type II diabetes mellitus [48]. We confirmed in this study that people with SCI showed increased risk factors for metabolic disease (systemic inflammation, hyperinsulinemia, lipidemia, and lactate) despite showing normal fasting glucose levels. We also demonstrated that a low frequency electrically induced exercise (LFE) reduced the insulin and glucose response at one hour after a glucose challenge in people with SCI. Our findings support that LFE after a meal may offer a daily life-style strategy to modulate peak insulin hormone release, without requiring high force contractions, which are known to be deleterious to osteoporotic bone in people with SCI. Future longitudinal investigations are warranted to determine if the effects demonstrated by LFE in this study are responsive when using a standard mixed meal rather than a highly concentrated glucose challenge; and to ascertain if LFE will longitudinally attenuate the development of diabetes among people with SCI.

Hypersecretion of insulin during a glucose tolerance test, independent of insulin resistance or blood glucose levels, may serve as an early predictor of those at risk for developing type II DM [17]. Consistent with other reports [13,49] our participants with SCI showed higher fasting insulin levels as compared to people without SCI. Further, after consuming glucose, the peak insulin for people with SCI was significantly higher than that observed among the healthy non-SCI participants [50]. The fasting hyperinsulinemia was coupled with “relatively” normal fasting glucose levels supporting that the “hyper-secretion” of insulin was important for metabolic homeostasis [17]. The timeline for chronic hyper-secretion of insulin, either during fasting or after a meal, to trigger “insulin resistance” is not precisely known. However, paralysis appears to promote a phenotype prone for hyper-secretion of insulin at rest and after ingestion of carbohydrates [23,51], which likely leads to insulin resistance and diabetes. Because hyper-secretion of insulin appears to be an important precursor to a “worse clinical and metabolic phenotype” it is important to identify lifestyle interventions that may attenuate the daily insulin spike and ultimately the progression to insulin resistance and diabetes among people with SCI [17].

An important discovery in this study is that insulin levels were blunted as an acute response to activating the skeletal muscle after the glucose challenge. The acute response of exercise, if repeated daily as a behavioral change in lifestyle, genetically promotes molecular pathways for hypertrophy and endurance [2,6,40,41,46,47]. Chronically increasing the oxidative capacity of muscle will enable the muscle to stay active longer without the buildup of lactate, perform more work, and ultimately have the capacity to consume more energy with each electrically induced contraction. Indeed, insulin receptors on oxidative muscle are purported to be more sensitive to insulin as compared to those on glycolytic muscle [52]. Taken together, the acute response of this protocol on insulin, if transitioned to a lifestyle behavior, may yield benefits from both acute and chronic signaling in paralyzed skeletal muscle. These findings also open the door to future studies to evaluate hybrid post prandial muscle activation by combining electrically induced exercise with volitional contractions of the upper extremities [8].

The population of people without SCI did not show attenuation of insulin with the LFE protocol. This may be because they had normal fasting insulin levels and were regular exercisers (activity levels, BMI) with muscle already saturated with adaptations for insulin/glucose homeostasis. The regular use of their thigh muscles, even in walking to the lab for the study, as compared to people with SCI in wheelchairs may explain this discrepancy. The LFE did induce an increase in HR for both populations supporting that there was increased muscle work with a systemic metabolic challenge induced by the stimulation protocol [18]. The nearly 3-fold increase in lactate for the SCI population, as compared to the non-SCI population, supports our contention that greater “anaerobic glycolysis” was triggered in the SCI population. This would be expected given the previous verification that most paralyzed muscle is comprised of glycolytic, fast, fatigable fibers. Furthermore, as expected, the people without SCI showed limited increase in lactate, which may eliminate another signaling source for an increase in insulin from the pancreas [17].

Our finding that people with SCI showed increased CRP, indicative of systemic inflammation is congruent with other reports [53]. At baseline, CRP was above the threshold for classifying individuals at high risk for cardiovascular disease [53]. We also discovered elevated serum uric acid (UA) at baseline. It is known that serum UA levels co-vary with insulin levels, insulin resistance, and pre-diabetes in people without SCI [54]. As expected, alkaline phosphatase (ALP) was elevated in our SCI population, which supports bone turnover and the significant osteoporosis among people with SCI [55]. Taken together, these baseline findings support that even people with SCI who are considered “healthy” have many of the characteristic precursors consistent with the development of cardiometabolic and inflammatory diseases. Indeed, we were successful in acquiring a population of people with SCI who did not have elevated fasting glucose, but elevated insulin, C-reactive protein, and UA. Hence, we believe that we were successfully studying participants who are on a pathway to developing metabolic disease, and, for whom, may be responsive to preventive lifestyle rehabilitation recommendations.

### 4.1. Low Force Electrically Induced Exercise

A novel finding from this study is that we were able to use a low frequency (3 Hz) and low force (<10% max torque) electrically induced exercise (LFE) [6,56] to presumably trigger AMPK to GLUT4 signaling and decrease peak insulin. This finding is important because higher frequencies of stimulation are untenable for people living with such a severe level of osteoporosis [45]. The acute impact of low frequency and low force exercise on insulin during an OGTT provides the first demonstration of the utility of manipulating the frequency, intensity, and duration of a dose of electrical stimulation to promote a healthy intervention for people with paralyzed muscle. Importantly, this intervention is cost effective and has the potential to translate seamlessly into the clinic if confirmed by larger, longitudinal clinical trials.

### 4.2. Study Limitations

One limitation of our study is that we performed an OGTT with glucose rather than a mixed meal, which may not resemble the typical meal of people with SCI. Although the oral glucose tolerance test is moderately correlated to the mixed-meal tolerance test [48] in people without SCI, we must use caution in not over generalizing our findings. Another consideration in our study is that we did not have the power to stratify our participants by lesion level and we were slightly underpowered given the variation in glucose Although we observed no correlation between lesion level and insulin response, a larger sample size stratified by lesion level would be recommended in future studies.

## 5. Conclusions

People with SCI are unable to volitionally exercise their paralyzed muscles. In this study, we show that a low force, electrically induced bout of exercise acutely attenuates the insulin response in people with SCI one hour after glucose ingestion. While further research is needed to determine the long-term viability of electrically induced exercise after a standard meal, this study suggests that LFE may be a feasible lifestyle strategy to decrease post-prandial hyperinsulinemia and, perhaps, someday influence the development of type II diabetes among people with SCI.

## Figures and Tables

**Table 1:** Baseline demographics and blood biomarkers.

	Able-Bodied Mean ± SD *n* = 23	Spinal Cord Injured Mean ± SD *n* = 18	
Age (years)	27.0 ± 5.4	37.7 ± 13.7	†
Years Post Injury		10.7 ± 8.6	
Height (cm)	174.9 ± 11.5	183.9 ± 6.4	†
Mass (kg)	71.8 ± 14.7	86.3 ± 21.0	†
BMI (kg/m^2^)	23.7 ± 2.5	25.4 ± 5.4	
Glycemic Biomarkers			
Glucose (mg/dL)	91.6 ± 7.6	91.2 ± 12.3	
Insulin (µUI/L)	6.8 ± 3.1	16.0 ± 13.6	†
G:I Ratio	15.9 ± 6.3	9.4 ± 6.0	†
Lactate (mmol/L)	1.0 ± 0.4	1.3 ± 0.6	†
Inflammatory Biomarkers			
C-Reactive Protein (mg/L)	1.1 ± 1.3	7.8 ± 8.2	†
Uric Acid (mg/dL)	4.6 ± 1.7	6.0 ± 2.1	†
Alkaline Phosphatase (UI/mL)	52.9 ± 15.1	77.5 ± 17.8	†
Lipid Biomarkers			
Cholesterol (mg/dL)	178.4 ± 25.1	185.8 ± 40.5	
Low-Density Lipoprotein (mg/dL)	111.4 ± 26.6	140.7 ± 38.4	†
High-Density Lipoprotein (mg/dL)	65.0 ± 16.9	41.2 ± 11.5	†
Triglycerides (mg/dL)	92.5 ± 53.4	128.9 ± 102.2	

† *t*-test between AB and SCI, *p* < 0.05.

## Data Availability

The data presented in this study are available on request from the corresponding author.
